# Current Treatment of Melanoma Brain Metastases

**DOI:** 10.3390/cancers15164088

**Published:** 2023-08-14

**Authors:** Agnieszka Nowacka, Anna Fajkiel-Madajczyk, Jakub Ohla, Kamila Woźniak-Dąbrowska, Sara Liss, Karol Gryczka, Wojciech Smuczyński, Ewa Ziółkowska, Dominika Bożiłow, Maciej Śniegocki, Michał Wiciński

**Affiliations:** 1 Department of Neurosurgery, Nicolas Copernicus University in Toruń, Collegium Medicum in Bydgoszcz, ul. Curie Skłodowskiej 9, 85-094 Bydgoszcz, Poland; k.wozniak@cm.umk.pl (K.W.-D.); sara-liss@wp.pl (S.L.); sniegocki@cm.umk.pl (M.Ś.); 2 Department of Pharmacology and Therapeutics, Nicolas Copernicus University in Toruń, Collegium Medicum in Bydgoszcz, ul. Curie Skłodowskiej 9, 85-090 Bydgoszcz, Poland; aniafajkiel1@gmail.com (A.F.-M.); 98.karol@wp.pl (K.G.); michal.wicinski@cm.umk.pl (M.W.); 3 Department of Orthopaedics and Traumatology, Nicolas Copernicus University in Toruń, Collegium Medicum in Bydgoszcz, ul. Curie Skłodowskiej 9, 85-094 Bydgoszcz, Poland; jakub.ohla@wp.pl; 4 Department of Physiotherapy, Nicolas Copernicus University in Toruń, Collegium Medicum in Bydgoszcz, ul. Techników 3, 85-801 Bydgoszcz, Poland; w.smuczynski@cm.umk.pl; 5 Department of Pediatrics, Washington University School of Medicine, St. Louis, MO 63110, USA; eziolkowska@wustl.edu; 6 Anaesthesiology and Intensive Care Clinical Ward, The 10th Military Research Hospital and Polyclinic, ul. Powstańców Warszawy 5, 85-681 Bydgoszcz, Poland; bozilow@wp.pl

**Keywords:** melanoma, melanoma brain metastases, MBM, BRAF mutation, immunotherapy, anti-PD-1, anti-CTLA-4, targeted therapy, BRAF and MEK inhibitors

## Abstract

**Simple Summary:**

Melanoma has the highest mortality rate of all skin cancers and its prognosis is worsened by metastasis to the brain. Before discovering new treatments for metastases, the prognosis of patients was poor. Currently, research is being conducted on new drugs using immunotherapy (immune checkpoint inhibitors: anti-PD-1, anti-CTLA-4) and targeted therapy (BRAF and MEK inhibitors) to improve the prognosis of patients. In this article, we summarize the current state of knowledge about the effects of treating brain metastases with new systemic therapies.

**Abstract:**

Melanoma is a type of skin cancer in which there is a strong correlation between its occurrence and exposure to ultraviolet radiation. Although it is not the most common skin cancer, it has the highest mortality rate of all skin cancers. The prognosis of patients is significantly worsened by melanoma metastasis to the brain, which often occurs in patients with advanced disease. The formation and development of melanoma metastases to the brain involve a very complex process, and their mechanisms are not fully understood. One of the ways for metastatic melanoma cells to survive and develop cancer in the brain environment is the presence of oncogenic BRAF mutation, which occurs in up to 50% of metastatic melanoma cases. Before discovering new methods of treating metastases, the overall survival of patients with this disease was 6 months. Currently, research is being conducted on new drugs using immunotherapy (immune checkpoint inhibitors: anti-PD-1, anti-CTLA-4) and targeted therapy (BRAF and MEK inhibitors) to improve the prognosis of patients. In this article, we summarize the current state of knowledge about the results of treating brain metastases with new systemic therapies.

## 1. Introduction

The term melanoma is derived from Greek and was first used by Hippocrates to describe a black tumor on the skin [1]. Melanoma originates from melanocytes that have undergone malignant transformation [2,3,4], and these cells are most abundant in the basal layer of epidermis, which is why cutaneous melanoma is the most common type. However, this neoplasm can also be found in less obvious locations, such as the uvea, gastrointestinal tract, genitourinary tract and the meninges [3,5,6].

Melanoma usually starts as a flat or slightly raised pigmented lesion that spreads horizontally across the skin, hence the name of the most common subtype-superficial spreading melanoma, which accounts for approximately 70% of cases [7]. The most significant risk factor for melanogenesis appears to be exposure to ultraviolet radiation (from both natural and artificial sources) [8,9]. Among many others, the presence of multiple and/or dysplastic nevi, male sex, Caucasian origin and geographical location are of particular relevance. The highest incidence of melanoma is observed in Australia, New Zealand and Scandinavia [10].

Although melanoma is less prevalent than other skin cancers, it has the highest fatality rate [11,12]. The reasons for this include diagnostic difficulties, rapid growth, a tendency to spread locally and distantly, and resistance to treatment. The incidence of melanoma has been on the rise in many countries in recent decades. According to GLOBOCAN Statistics, about 324,635 new cases of cutaneous melanoma were reported worldwide in 2020, and 57,043 people died from this disease [13]. The American Cancer Society estimates that approximately 97,610 new cases of melanoma will be diagnosed and 7990 people will die from melanoma in the United States in 2023 [14].

Malignant melanoma appears to be a significant public health problem. The management of patients with melanoma requires a multidisciplinary approach, involving primary care physicians, dermatologists, clinical oncologists and surgeons. One of the most interesting and challenging issues regarding melanoma treatment today is the management of melanoma metastases in the brain. It is worth noting that melanoma is the third most common source of intracranial metastases in adults following lung and breast cancer [15,16]. Up to 60% of patients with advanced melanoma develop brain metastases during the course of their disease [17,18,19], and the presence of brain metastases significantly worsens a patient’s prognosis [20]. Before the era of new therapies, the overall survival of patients with melanoma brain metastases was usually no longer than 6 months [19,21,22].

The migration of melanoma cells to the brain parenchyma is a complex process, in which perhaps the most important step is the disruption of the integrity of the blood-brain barrier. The role of the complex interactions between the brain microenvironment and melanoma cells is also in the spotlight [18,23]. The depth of invasion at the primary site is assessed by the m Breslow scale, male sex, high serum lactate dehydrogenase (LDH) level, head or neck as the primary site, and visceral or nodal involvement, all of which are indicated as risk factors for the development of melanoma brain metastases [4,24,25]. About 80% of melanoma brain metastases are located supratentorial, and there is often more than one lesion at a time [19,26]. What is interesting is that some authors also suggest that melanoma brain metastases have a strong tendency to bleed and trigger seizures [27,28].

Brain metastases are a significant clinical problem, but the last decade has seen great progress in the development of treatment strategies for advanced melanoma. Today, the contribution of immunohistochemistry and the genetic analysis of tumors to the diagnostic process is becoming increasingly important. Melanoma is one of the most immunogenic cancers and one of the tumors with the highest mutational burden [29,30,31]. Molecular profiling plays a key role in the management of malignant melanoma, particularly in the search for new drug targets and the possible personalization of therapy. So far, at least 20 genes have been identified that play a role in melanoma pathogenesis. The most frequently mutated are BRAF, NRAS and KIT oncogenes [29,32,33].

There are currently three different groups of treatment available for melanoma brain metastases: neurosurgical resection of lesions, radiotherapy (whole-brain or stereotactic) and new systemic therapies. The latter category includes two main classes of agents. The first, known as immune checkpoint inhibitors (ICIs), antagonize cytotoxic T-cell antigen 4 (CTLA-4) or programmed cell death protein 1 (PD-1). The second are drugs targeted against key protein kinases in melanoma pathogenesis, such as BRAF and MEK.

The aim of this article is to summarize the current state of knowledge in the field of treating melanoma brain metastases using immunotherapy as well as targeted therapies.

## 2. Biology of Melanoma Brain Metastases

The mechanisms of the formation and development of melanoma metastases (MMs) in the brain parenchyma are not fully understood, however, the expression of the proteins involved in the process of neurodegeneration is different in melanoma cells explanted from brain metastates and extracranial metastasis cells [34]. 

The dissemination of melanoma proceeds through the bloodstream and the condition for MMs to develop in the brain is crossing the blood-brain barrier (BBB) [35]. The structure of the endothelial cells (ECs) located in the brain vessels and the presence of the pericites and foot processes (FPs) of the astrocytes in their vicinity are of key importance for maintaining the integrity of the BBB [35]. Pericites are cells embedded in the basement membrane (BM) outside the lumen of the vessel that surround it in a circular manner [36], while the astrocytes contact the outer part of the BM by FPs [37]. ECs form a compact structure connected by tight junctions (TJs) and adherens junctions [38], and are characterized by a low level of transcytosis [39], high expression of ion channels and functional variability regulated by astrocytes [35]. The role of pericytes is to regulate the junctions between endothelial cells and the process of transcytosis [37]. Crossing the BBB by the metastatic melanoma cells (MMCs) is a complex and multi-stage process, the essence of which is the loosening and widening of the tight connections of ECs and damage to the BM with the participation of proteases, i.e., MMP-9 and heparanase. The damage to the connections between the ECs is preceded by the processes of cerebral vasodilation, increased secretion of VEGF and decreased expression of ZO-1 (*zonula occludens protein 1*) [35]. The ZO family proteins play an important role in the formation of functional TJs by cross-linking and binding to various TJs proteins and anchoring them to the cytoskeleton [40]. The compounds secreted by MMCs that serve to loosen the TJs between the ECs are angiopoetin-2 and numerous pro-invasive integrins (α3β1, αvβ3 and α4β1) [35]. The process of the dissemination of MMCs in the brain, after penetrating the BBB, initially proceeds along the vessels, where micrometastases are formed surrounding the vessels, which eventually form metastatic tumors [35].

While the brain environment is generally hostile to MMCs as most of them die after crossing the BBB, brain cells have both a supportive and suppressive effect on their development [35]. The mechanisms ultimately determining the development of a metastatic melanoma tumor in the brain are not known, but this condition is preceded by a set of complex interactions between MMCs and glial cells, mainly microglia, oligodendrocytes and astrocytes [35]. The most far-reaching interactions connect MMCs with astrocytes [35]. The protein necessary for the survival and development of melanoma metastases in the brain parenchyma is amyloid beta (Aβ), which activates the surrounding astrocytes to differentiate into a favorable, prometastatic phenotype, protecting MMCs from microglial phagocytosis [34]. 

Genetic and functional changes are observed in MMCs, enabling them to survive and develop in the brain environment. The most common oncogenic mutations in melanoma cells are BRAF and NRAS mutations [41]. The BRAF mutation affects serine-threonine kinase BRAF and is found in ~40–60% of melanoma [42], while the NRAS mutation affects the RAS protein responsible for activating the signaling pathways that control cell proliferation, differentiation and survival [43]. The NRAS mutation is found in ~25% of melanoma cases [44]. The key suppressor genes that are dysregulated in MMCs by genetic or epigenetic mechanisms are PTEN (*Phosphatase* and *Tensin Homolog*) and p16 [45], however, it should be emphasized that gene expression differences are observed between melonoma brain metastases (MBMs) and extracranial melanoma metastases [46]. An important disorder promoting the development of melanoma metastases (MMs) is the hyperactivation of the PI3K/AKT pathway as a result of the loss of the PI3K activation inhibitor PTEN [41]. The loss of PTEN is characteristic of MMCs in the brain, and it occurs only in the presence of astrocytes in the mechanism of the secretion of microRNA-containing exosomes [47]. The excessive activity of the PI3K/AKT pathway triggers the activation of the mTOR (*mammalian Target of Rapamycin*) pathway [41], which is a serine/threonine kinase consisting of two different multicomponent mTORC1 and mTORC2 interacting complexes. These complexes, through signaling pathways, affect cell growth and proliferation, their metabolism, protein synthesis and motility [48]. In tumors of various origins, the activation of the PI3K/Akt/mTOR pathway is associated with cell proliferation and angiogenesis, which results in invasiveness and disease progression [49]. It is probable that the oncogenic BRAF mutation and activation of the PI3K/AKT pathway occur together and are important in the formation of MMs in the brain, with other mechanisms underlying the activation of this pathway in BRAF and NRAS mutations [41]. Furthermore, factors secreted by astrocytes, such as neurotrophins, interleukins (IL-6, IL-8) and G-CSF, are responsible for the hyperactivation of the PI3K/AKT pathway in MMCs [41]. 

The number of MBMs and their carcinogenicity increases due to the expression of CCR4 [41]. Klein et al. showed that the overexpression of CCR4 increases the invasiveness of MMs and is associated with an increased number of brain micrometastases [50]. Additionally, CCL17 and CCL22 are CCR4 ligands and are secreted by astrocytes and microglial cells [41].

Based on the available literature, it seems that the mechanisms described above play a major role in the dissemination and development of MBMs.

## 3. MBM Treatment

Immune checkpoint inhibitors (ICIs), involving monoclonal antibodies, are a type of novel anti-cancer therapy targeting programmed cell death protein 1 (PD-1), its ligand PD-L1 and cytotoxic T-lymphocyte-associated protein 4 (CTLA-4) [51]. PD-1 is located on T lymphocytes and its interaction with PD-L1, as expressed on the surface of tumor cells, causes apoptosis of cytotoxic T lymphocytes while preventing apoptosis of Treg cells [52]. CTLA-4 is a co-stimulatory protein that interacts with the receptors on T lymphocytes to inhibit effector T cells [53].

The concept of the immune checkpoint blockade (ICB) in cancer treatment was developed by Jim Allison and his colleagues. They demonstrated that antibodies blocking the T-cell co-inhibitory receptor CTLA-4 can induce tumor regression in mice [54]. This discovery and further research led to the approval of the first agent used for metastatic melanoma-ipilimumab (FDA, 2011) [51]. Immunotherapy is the primary management option for melanoma patients with CNS metastases in the absence of the V600 mutation of the BRAF gene. In patients with a BRAF mutation, the decision to opt for immunotherapy or BRAFi with MEKi treatment depends on the clinical situation [55]. Numerous studies have proven that immunotherapy is safe and provides durable responses in melanoma brain metastases. Combination therapy (anti-CTLA-4 + anti-PD-1) increases response rates compared to monotherapy [56]. A summary of the most important clinical trials from recent years is presented in Table 1.

The effectiveness of nivolumab compared to nivolumab (anti-PD-1) + ipilimumab (anti-CTLA-4)—the most common combination in immunotherapy—in the treatment of melanoma brain metastases was assessed in a phase II randomized parallel-group clinical trial (NCT02374242). With a median follow-up of 17 months, 46% of patients in cohort A (nivolumab + ipilimumab), 20% in cohort B (nivolumab) and 6% in cohort C (nivolumab; patients with failed local therapy or with neurological symptoms or leptomeningeal disease) achieved intracranial responses. Complete intracranial responses occurred in 17% of patients in cohort A, 12% in cohort B and none in cohort C. Treatment-related adverse events (TRAEs) were most common in patients treated with nivolumab + ipilimumab (97% of patients vs. 68% in cohort B and 50% in cohort C). Grade 3 or 4 TRAEs also occurred most frequently in cohort A (54% vs. 16% and 13%, respectively). There were no treatment-related deaths. Both nivolumab in combination with ipilimumab and nivolumab alone are active in melanoma brain metastases, but a high percentage of patients achieved an intracranial response with the combination [57].

A further study that evaluated the effectiveness of the above combination (nivolumab + ipilimumab) in asymptomatic and symptomatic MBM was CheckMate 204. Intracranial clinical benefits were observed in 57.4% of patients in cohort A (asymptomatic patients) and 16.7% of patients in cohort B (symptomatic patients). An objective response was observed in 53.5% of patients (cohort A) and 16.7% of patients (cohort B). For patients in cohort A, both 36-month progression-free survival (PFS) and overall survival (OS) were higher than in cohort B (54.1% vs. 18.9% and 71.9% vs. 36.6%, respectively). The most common grade 3–4 TRAEs were increased alanine aminotransferase and aspartate aminotransferase (cohort A), but in cohort B, no grade 3 TRAEs occurred in more than one patient. The most common serious TRAEs were colitis, diarrhea, esophagitis and alanine aminotransferase elevations in cohort A. There was also one treatment-related death caused by myocarditis. No serious TRAEs occurred in more than one patient in cohort B. The final 3-year results of CheckMate 204 support the continued use of nivolumab 1 mg/kg + ipilimumab 3 mg/kg as the standard of first-line care in asymptomatic patients with MBM who are candidates for immunotherapy. This clinical trial showed that there is still an unmet need to implement new treatment regimens in symptomatic patients, as few patients responded to treatment with nivolumab + ipilimumab [58].

The NIBIT-M2 study was designed to test the combination of fotemustine (a nitrosourea alkylating agent) + ipilimumab and ipilimumab + nivolumab for melanoma metastases to the brain. The primary endpoint of the study was overall survival, and the results for the different groups are shown in Table 1. The median OS was highest for the combination of ipilimumab + nivolumab (29.2 months). Similarly, four-year survival was significantly higher for ipilimumab + nivolumab compared with fotemustine [(41.0%; 95% CI, 20.6–61.4) vs. 10.9% (95% CI, 0–24.4; *p* = 0.015)]. In contrast, for the ipilimumab + fotemustine combination, it was 10.3% (95% CI, 0–22.6). Grade 3–4 TRAEs occurred in 48% (fotemustine), 69% (ipilimumab + fotemustine) and 30% (ipilimumab + nivolumab), respectively. There was no treatment-related death. This is another study that has proven the efficacy of the ipilimumab + nivolumab treatment in melanoma patients with asymptomatic brain metastases. [59].

The NCT02085070 clinical trial conducted a long-term follow-up of patients treated with pembrolizumab (anti-PD-1) with new or progressive brain metastases. Pembrolizumab was administered for up to 24 months, and 26% of patients achieved a response to brain metastases. The median PFS and OS times were 2 and 17 months, respectively. At month 24 of the study, 11 patients (38%) were alive. Neurological adverse events occurred in 65% of patient, and all adverse events were grade 1 or 2 (except one). Pembrolizumab shows activity in melanoma brain metastases, acceptable toxicity and a long-term response [60].

A multicenter, open-label, parallel-group phase II study (MIRACULUM) evaluated the efficacy and safety of two dosing regimens of prolgolimab (anti-PD-1) in patients with advanced melanoma. An objective response was observed in 38.1% (arm 1:1 mg/kg every 2 weeks) and 28.6% (arm 2: 3 mg/kg every 3 weeks) of patients. Grade 3–4 TRAEs occurred in 12.7% and 3.2% of patients in arms 1 and 2, respectively. Two-year PFS was 33.3% (arm 1) and 30.2% (arm 2) and the 2-year OS was 57.1% and 46.0%, respectively. Prolgolimab shows significant anti-tumor activity and a good safety profile in patients with advanced melanoma [61].

With the development of immunotherapy, a new category of side effects—immune-related adverse events (irAEs)—have been observed, and they are slightly different from the side effects of “classic” cancer chemotherapy. The adverse events associated with ICI therapy most commonly affect the skin, gastrointestinal tract and endocrine glands, but can affect virtually any tissue [62,63,64,65]. They often occur shortly after starting treatment, but a small number of patients may develop irAEs months after finishing treatment [64]. These reactions are more frequent with combination therapy (e.g., anti-CTLA4 + anti-PD-1) than monotherapy [64,65]. 

The most common activating mutation found in melanoma is BRAF-V600E, which occurs in approximately 50% of metastatic melanoma [66]. When it is present, targeted therapies (TT) such as BRAF and MEK inhibitors (BRAFi/MEKi), which block the activated mitogen-activated protein kinase (MAPK) cascade, are most commonly used [67]. BRAF inhibitors include medications such as vemurafenib, dabrafenib and encorafenib, and MEK inhibitors include agents such as trametinib, cobimetinib and binimetinib [55]. These medications show superior efficacy compared to chemotherapy in melanoma [68,69] and, in addition, have shown high efficacy in melanoma with brain metastases, as demonstrated by clinical trials [70,71]. Numerous scientific discoveries have led to the design of clinical trials evaluating combinations of BRAFi/MEKi and immunotherapy, resulting in rapid (targeted therapy) and long-term (immunotherapy) responses in patients with metastatic melanoma [72,73].

An example of combination treatment using immunotherapy and targeted therapy is the TRICOTEL study. It showed that the addition of atezolizumab (anti-PD-L1) to vemurafenib (BRAFi) + cobimetinib (MEKi) provides promising intracranial activity in BRAFV600-mutant melanoma patients with CNS metastases. Patients received intravenous atezolizumab + oral cobimetinib (BRAFV600 wild-type cohort) and intravenous atezolizumab + oral vemurafenib + oral cobimetinib (BRAFV600 mutation-positive cohort). The median follow-up was 7–9 months and 2–6 months, respectively. The intracranial ORR was 42% by IRC assessment in the BRAFV600 mutation-positive cohort and 27% in the BRAFV600 wild-type cohort. Grade 3 or worse TRAEs occurred in 68% of patients who received atezolizumab + vemurafenib + cobimetinib. In this group, the most common adverse effects included increased lipase levels (25% of patients) and increased blood creatine phosphokinase levels (17% of patients). Moreover, 53% of patients treated with atezolizumab + cobimetinib experienced grade 3 or worse TRAEs—most commonly anemia (13% of patients) and acne dermatitis (13% of patients). Serious TREAs occurred in 23% of patients in the BRAFV600 mutation-positive cohort and 13% of patients in the BRAFV600 wild-type cohort. One death in the BRAFV600 mutation-positive cohort due to limbic encephalitis was considered to be related to the atezolizumab treatment [74].

Another study included 275 patients with BM. An efficacy analysis in a subgroup of melanoma patients with the BRAFV600 mutation and BM, who were treated with dabrafenib (BRAFi) + trametinib (MEKi) in an open-label, non-randomized phase IIIb study, showed that the serum lactate dehydrogenase (LDH) level, Eastern Cooperative Oncology Group (ECOG) status and number of metastatic sites at baseline were significant predictors of PFS in patients treated with dabrafenib + trametinib. The ORR was 41.5% and the median PFS was 5.68 months. There were 164 progression events—disease progression or death—during the study [75].

The aim of the BUMPER trial (phase II) was to evaluate the safety and efficacy of buparlisib in patients with pretreated, progressive MBM. This medication, which is a PI3K (Phosphoinositide 3-kinase) inhibitor, was used in patients with asymptomatic MBM who were unable to receive local treatment. These patients had progression under immunotherapy (presence of BRAF wild-type) and BRAFi/MEKi therapy (presence of BRAF-V600E). The primary endpoint assessed by the investigators was the intracranial disease control rate. The overall response rate, duration of the response of intracranial disease, overall response, PFS, OS and safety and tolerability of the agent were also assessed. The median PFS was 42 days and the median OS was 5.0 months. Buparlisib was well tolerated. No intracranial responses were observed, but this may have been due to the inclusion of only heavily treated patients in the study. In the case of the hyperactivation of the PI3K-AKT pathway in MBM patients, there is reason to seek PI3K inhibitor-based treatment combinations. Perhaps the use of PI3K inhibitors at earlier therapeutic lines can overcome primary resistance to immunotherapy [76].

A clinical trial called SECOMBIT [77] investigated the effectiveness of sequential immunotherapy and targeted therapy in treating patients with BRAFV600-mutant melanoma. The trial included three different treatment arms and aimed to assess overall survival (OS) at 2 years as the primary endpoint, along with several secondary endpoints, such as progression-free survival, 3-year OS, the response rate, the duration of the response and biomarkers. A total of 209 patients from 37 sites in 9 countries with untreated, metastatic BRAFV600-mutant melanoma were randomly assigned to one of the three treatment arms. Arm A received encorafenib and binimetinib until progressive disease (PD), followed by ipilimumab and nivolumab for four cycles, and then nivolumab every 2 weeks. Arm B received ipilimumab and nivolumab until PD, followed by encorafenib and binimetinib. Arm C received encorafenib and binimetinib for 8 weeks, followed by ipilimumab and nivolumab until PD, and then encorafenib and binimetinib again. The results showed that at a median follow-up of 32.2 months, the median OS was not reached in any of the treatment arms, indicating that the patients in all arms showed longer survival than expected. The 2-year and 3-year OS rates were 65% (95% CI, 54 to 76) and 54% (95% CI, 41 to 67) in arm A, 73% (95% CI, 62 to 84) and 62% (95% CI, 48 to 76) in arm B, and 69% (95% CI, 59 to 80) and 60% (95% CI, 58 to 72) in arm C. These results demonstrated clinically meaningful survival benefits for patients with BRAFV600-mutant melanoma. The trial also assessed safety throughout the sequential treatment in all participants who received at least one dose of the study medication. No new safety concerns were identified. In conclusion, sequential immunotherapy and targeted therapy in patients with BRAFV600-mutant melanoma, as evaluated in the SECOMBIT trial, provided significant survival benefits. These findings support the use of this treatment approach for improving outcomes in this patient population.

Both combination programmed cell death protein 1/cytotoxic T-cell lymphocyte-4 (PD-1/CTLA-4) blockade and dual BRAF/MEK inhibition have demonstrated significant clinical benefits in patients with metastatic melanoma harboring BRAFV600 mutations, leading to widespread regulatory approval [78]. However, there is a lack of prospective data to guide the selection of the initial therapy or treatment sequence in this patient population. The study aimed to determine the optimal efficacy of different initial treatments and treatment sequences. In a phase III trial, treatment-naive patients with BRAFV600-mutant metastatic melanoma were randomly assigned to receive either combination therapy with nivolumab/ipilimumab (arm A) or dabrafenib/trametinib (arm B) in the first step. Upon disease progression, patients were enrolled in the second step to receive the alternate therapy: dabrafenib/trametinib (arm C) or nivolumab/ipilimumab (arm D). The primary endpoint was overall survival (OS) at 2 years, and secondary endpoints included OS at 3 years, an objective response rate, response duration, progression-free survival, crossover feasibility and safety. A total of 265 patients were enrolled, and 73 patients proceeded to the second step (27 in arm C and 46 in arm D). The study was halted prematurely by the independent Data Safety Monitoring Committee due to the achievement of a clinically significant endpoint. The 2-year OS rate for patients starting with arm A was 71.8% (95% CI, 62.5 to 79.1), whereas it was 51.5% (95% CI, 41.7 to 60.4) for arm B (log-rank *p* = 0.010). Progression-free survival in the first step favored arm A (*p* = 0.054). Objective response rates were as follows: arm A, 46.0%; arm B, 43.0%; arm C, 47.8%; and arm D, 29.6%. The median duration of response was not reached for arm A, while it was 12.7 months for arm B (*p* < 0.001). Approximately 52% of patients with documented disease progression crossed over to the alternate therapy. Grade ≥ 3 toxicities occurred with a similar frequency across the arms, and the toxicity profiles of the regimens were as expected. Based on the study findings, the preferred treatment sequence for the majority of patients should involve combination therapy with nivolumab/ipilimumab, followed by BRAF and MEK inhibitor therapy, if needed.

## 4. Conclusions

Melanoma remains a major public health concern as it is a cancer with a high mortality rate. Melanoma is associated, among others, with diagnostic difficulties, rapid growth, as well as local and distant metastases. One of the biggest problems faced by melanoma patients are brain metastases, which occur in up to 60% of patients with advanced melanoma, and the presence of brain metastases significantly worsens the prognosis of patients. Before discovering new methods of treating metastases, the overall survival of patients with this disease was 6 months. An alternative to their standard treatment with neurosurgery and radiotherapy are new systemic therapies using immunotherapy and molecularly targeted treatment. Studies conducted in recent years have shown that combination therapy with the use of immune checkpoint inhibitors-anti-PD-1 (nivolumab) and anti-CTLA-4 (ipilimumab) brings better results than their use in monotherapy. In melanoma patients, the most common oncogenic mutation is the BRAF-V600E mutation, which occurs in up to 50% of metastatic melanomas, and these patients should be treated with targeted therapies. One of the studies showed good results of combination therapy with immunotherapy methods-atezolizumab (anti-PD-1) and targeted therapy-vemurafenib (BRAFi) and comimetinib (MEKi). Despite the promising results and improved survival of patients treated with new methods, these therapies are not without side effects, so further research is still needed in this area. However, it seems that new systemic therapies have the chance to play a significant role in the future as a first-line drug in the treatment of melanoma metastases to the brain.

## Figures and Tables

**Table 1:** Clinical trials of immunotherapy in melanoma brain metastases.

Clinical Trial	Strategy	Clinical Trial Phase	Patient Characteristics	Number of Patients Enrolled	Intervention	mPFS (Months)	mOS (Months)	Ref.
NCT02374242	Nivolumab + Ipilimumab or Nivolumab	II	Patients with asymptomatic (cohort A, B) and symptomatic (cohort C) MBM.	79	Intravenous nivolumab 1 mg/kg + ipilimumab 3 mg/kg every 3 weeks for four doses, then nivolumab 3 mg/kg every 2 weeks (cohort A); intravenous nivolumab 3 mg/kg every 2 weeks (cohort B and C).	NR (cohort A) 2.5 (cohort B) 23 (cohort C)	NR (cohort A) 18.5 (cohort B) 5.1 (cohort C)	[57]
NCT02320058	Nivolumab + Ipilimumab	II	Patients with asymptomatic (cohort A) and symptomatic (cohort B) MBM.	119	Intravenous nivolumab 1 mg/kg + ipilimumab 3 mg/kg every 3 weeks for four doses, followed by nivolumab 3 mg/kg every 2 weeks for up to 2 years (until disease progression or unacceptable toxicity).	NR	NR	[58]
NCT02460068	Fotemustine or Ipilimumab + Fotemustine or Ipilimumab + Nivolumab	III	Patients ≥ 18 years of age with *BRAF* wild-type or mutant melanoma, and active, untreated, asymptomatic BM.	27 (fotemustine) 26 (fotemustine + ipilimumab) 27 (ipilimumab +nivolumab)	Fotemustine 100 mg/m^2^ over 60 min, once every week for three doses (weeks 1, 2 and 3; induction phase), and once every 3 weeks from week 9 for six doses (maintenance phase). Fotemustine + ipilimumab 10 mg/kg over 90 min given as induction every 3 weeks for four doses (weeks 1, 4, 7 and 10), and then every 12 weeks from week 24 (maintenance phase). Ipilimumab 3 mg/kg over 90 min together with nivolumab 1 mg/kg over 60 min every 3 weeks for four doses (weeks 1, 4, 7 and 10; induction phase), and from week 12 nivolumab 3 mg/kg over 60 min every 2 weeks (maintenance treatment).	3.0 (fotemustine) 3.3 (ipilimumab+ fotemustine) 8.7 (ipilimumab+ nivolumab)	8.5 (fotemustine) 8.2 (ipilimumab+ fotemustine) 29.2 (ipilimumab+ nivolumab)	[59]
NCT02085070	Pembrolizumab	II	Patients with melanoma with one or more asymptomatic, untreated 5–20 mm brain metastases not requiring corticosteroids; 70% of patients-prior systemic therapy.	23	10 mg/kg intravenously every 2 weeks for up to 24 months.	2	17	[60]
NCT03269565	Prolgolimab	II	Patients with advanced cutaneous or non-cutaneous melanoma, including stable brain metastasis, without autoimmune disease; no prior targeted therapy.	126	1 mg/kg every 2 weeks (arm 1) or 3 mg/kg every 3 weeks (arm 2) until disease progression or intolerable toxicity.	6.6 (arm 1) 3.7 (arm 2)	NR (arm 1) 15 (arm 2)	[61]

NR—not reached; mPFS—median progression-free survival; mOS—median overall survival.
